# Membrane Palmitoylated Protein 7 is Required for Osteogenesis and is Linked with Bone Mineralization and Osteoporosis: The Functional Evaluation of GEFOS GWAS Hit

**DOI:** 10.1007/s00223-025-01439-w

**Published:** 2025-10-17

**Authors:** Petra Malavašič, Jasna Lojk, Marija Nika Lovšin, Radko Komadina, Gregor Haring, Rihard Trebše, Fernando Rivadeneira, David Karasik, Barbara Ostanek, Janja Marc

**Affiliations:** 1 Department of Laboratory Diagnostics, Novo Mesto General Hospital, Novo Mesto, Slovenia; 2 Faculty of Pharmacy, University of Ljubljana, Aškerčeva Cesta 7, 1000 Ljubljana, Slovenia; 3 Clinical Institute of Clinical Chemistry and Biochemistry, University Medical Centre Ljubljana, Ljubljana, Slovenia; 4 Department for Research and Education, General Hospital Celje, Celje, Slovenia; 5 Institute of Forensic Medicine, University of Ljubljana, Ljubljana, Slovenia; 6 Orthopaedic Hospital Valdoltra, Ankaran, Slovenia; 7 Faculty of Medicine, University of Ljubljana, Ljubljana, Slovenia; 8 Erasmus University Medical Center, Rotterdam, The Netherlands; 9 The Azrieli Faculty of Medicine, Bar-Ilan University, Safed, Israel

**Keywords:** *MPP7*, HOS cell lineage, Human bone tissue, Adipogenesis, Human muscle tissue

## Abstract

Genome-wide association studies have identified multiple loci associated with bone mineral density, a major determinant of osteoporotic fracture risk. At one such locus, genetic, bioinformatic, and zebrafish knockout data strongly prioritize membrane palmitoylated protein 7 (MPP7) as a candidate gene, although its precise role in bone biology remains poorly defined. MPP7 encodes a member of the p55 Stardust family of membrane-associated guanylate kinase proteins, which are key regulators of epithelial cell polarity and junctional organization. Here, we investigated the functional role of MPP7 in bone biology. We found that MPP7 expression was significantly reduced—by approximately twofold—in bone tissue from osteoporotic patients compared with osteoarthritic patients and non-osteoporotic controls. Furthermore, we generated a CRISPR/Cas9-mediated MPP7 knockout in the human osteosarcoma HOS cell line and demonstrated that MPP7 deletion impairs osteogenic differentiation and completely abrogates mineralization through downregulation of ALPL expression. Knockout cells also displayed altered morphology, suggesting that MPP7 influences osteoblast function via effects on cell polarity and adhesion. Collectively, our findings, together with zebrafish genetic evidence, indicate that MPP7 plays a critical role in osteoblast differentiation and mineralization and may contribute to osteoporosis susceptibility in humans.

**Supplementary Information:**

The online version contains supplementary material available at 10.1007/s00223-025-01439-w.

##  Lay Summary

Osteoporosis causes bones to become fragile and more likely to break. We studied the gene *MPP7*, which has been linked to bone mineral density in genetic studies, but whose role in bone biology is unknown. We found that *MPP7* levels are lower in bone from osteoporotic patients. When we deleted this gene in bone-forming cells, they were no longer able to initiate mineralization. The cells also changed shape, suggesting that *MPP7* helps control their structure and function. These findings show that *MPP7* is important for the mineralization process and may be a potential target for treating osteoporosis.

## Introduction

Osteoporosis (OP) is the most common skeletal disorder in humans, characterized by decreased bone mineral density (BMD), reduced bone strength, and an increased risk of fractures. It affects more than one-third of postmenopausal women and, to a lesser extent, older men [1]. OP is asymptomatic in itself and is typically diagnosed only after a low-energy fracture, which is often followed by immobility or reduced physical activity, increased morbidity and mortality, and significant changes in patients’ lifestyles [2–4]. As such, OP substantially reduces quality of life, may lead to loss of independence, and poses a major healthcare and economic burden. With the aging global population, there is an urgent need for improved prevention strategies, early diagnostics, and novel treatment options.

Low BMD—the diagnostic marker of OP [5], is highly heritable and polygenic in nature, with heritability estimates ranging from 0.6 to 0.85 [6–8]. To date, genome-wide association studies (GWAS) and their meta-analyses have identified numerous loci associated with BMD [9–15]. However, the roles of many candidate genes within these loci in bone physiology remain largely uncharacterized. One such gene, located on chromosome 10, is *membrane palmitoylated protein 7* (*MPP7*; also known as MAGUK p55 subfamily member 7), which has been associated with heel [14, 16–18] and lumbar spine [10, 19] BMD measurements. GWAS have identified the *MPP7* locus (10p11.23) as being significantly associated with estimated bone mineral density (eBMD) (rs3905706) [10]. In addition, site specificity has been reported: *MPP7* is associated with lumbar spine but not femoral neck BMD, consistent with trabecular versus cortical bone differences between these sites [15]. Beyond bone phenotypes, *MPP7* has been robustly associated with lean mass in a large GWAS (*n* = 683,692, *p* = 1.5 × 10^−8^), pointing to a broader musculoskeletal role that further motivated its investigation in the context of bone–muscle cross-talk [19, 20]. Skeletal functional evidence is also available, as knockdown of MPP7 in zebrafish resulted in skeletal abnormalities, including impaired vertebral mineralization, which were partially rescued by human *MPP7* mRNA [21]. Furthermore, *MPP7* is constitutively expressed in human bone-derived cells during osteogenesis localizing to the plasma membrane and cytoplasm in osteoblasts [21]. Taken together, these data provide strong support for prioritizing *MPP7* at this locus, although contribution of nearby genes cannot be completely excluded. *MPP7* is a recently identified member of the membrane-associated guanylate kinase (MAGUK) family [22], primarily known for its role in membrane polarization and cell attachment. It interacts with LIN7A/C and DLG1 to form the *MPP7*–DLG1–LIN7A/C complex [23, 24], which co-localizes with E-cadherin at cell–cell junctions and is essential for tight junction formation [24, 25]. Similarly, *MPP7* interacts with CRB3 and PALS1 (MPP5) to form the DLG1–*MPP7*–CRB3–MPP5 complex, which also localizes to adherens and tight junctions [25]. Interestingly, both *DLG1* [16–18] and LIN7C [10, 15, 17, 18, 26] have been associated with BMD in multiple GWAS, highlighting the importance of cell adhesion and polarity in the regulation of BMD. Beyond its structural role, *MPP7* is involved in regulating cell proliferation and stem cell fate during tissue regeneration. By forming a complex with angiomotin (AMOT) and yes-associated protein 1 (YAP1) following cell detachment, *MPP7* modulates the entry of satellite cells into the cell cycle. Silencing *MPP7* in muscle satellite cells reduces proliferation and forces premature differentiation, thereby depleting the satellite cell pool [27, 28].

Adequate proliferation, stem cell renewal, and osteogenic differentiation are central to bone homeostasis and skeletal integrity. Osteoblasts, the bone-forming cells, differentiate from mesenchymal stem cells (MSCs), which also give rise to myoblasts, chondrocytes, and adipocytes [29]. MSC commitment is governed by tightly regulated signaling pathways, particularly the Wnt and Hippo pathways, along with lineage-specific transcription factors that orchestrate terminal differentiation and functional maturation, including mineralization. To date, only one prior functional study used morpholino-mediated knockdown of *MPP7* in zebrafish embryos, resulting in > 70% transcript suppression. The knockdown animals exhibited a characteristic ventral bending of the tail, impaired swimming ability, and markedly reduced vertebral bone mass and mineralization compared with wild-type (WT) controls and a pronounced deficit in vertebral mineralization. Co-injection of human *MPP7* mRNA partially rescued the phenotype, reinforcing the specificity of the observed skeletal defect. These findings constitute the first in vivo validation of *MPP7*’s role in bone development [21].

The aim of this study was to validate the involvement of *MPP7* in bone biology and to explore the underlying mechanisms by which it may influence BMD. We investigated whether *MPP7* could serve as a potential therapeutic target or biomarker in bone diseases. Here, we report that *MPP7* expression is reduced in osteoporotic bone and in muscle tissue from both osteoporotic and osteoarthritic patients compared to healthy controls. Furthermore, using CRISPR/Cas9-mediated *MPP7* knockout (KO) in the human osteosarcoma HOS cell line, we demonstrate that *MPP7* is critical for mineralization via regulation of *ALPL* expression. Our findings suggest that *MPP7* is involved in the regulation of bone mineralization and may contribute to the development of OP.

## Materials and Methods

### Bioinformatic Tools for *MPP7* Evaluation

To evaluate the functional relevance of *MPP7* at the 10p11.23 locus, we performed in silico analyses using publicly available GWAS summary statistics for BMD. Bayesian analysis (HuGE calculator) [30] was used to compute Bayes Factors for causality, while Gene Sifter (Knowledge Portal, Gene Sifter) was applied to assess tissue-specific expression enrichment. In addition, the Gene Effector Index (GEI) [31] was used to integrate GWAS, chromatin, and eQTL data and estimate the probability of *MPP7* as the effector gene at this locus.

### Bone and Muscle Tissue Collection

Human bone and muscle tissue samples were collected from Slovenian patients with osteoarthritis (OA) and OP, as previously described [32]. Bone tissue samples (intertrochanteric region of the proximal femur) and skeletal muscle samples (gluteus medius) were obtained from 84 individuals undergoing hip arthroplasty—37 with OA and 47 with OP—at the Department of Traumatology, General Hospital Celje, and the Orthopedic Hospital Valdoltra. BMD of the contralateral hip and lumbar spine was assessed using dual-energy X-ray absorptiometry (DEXA; Hologic QDR-1000, Waltham, MA, USA) either before or after surgery. OP was diagnosed clinically since all patients sustained hip fractures; we also measured BMD at the hip, femoral neck, and lumbar spine by DXA, according to the WHO definition (*t*-score ≤ − 2.5). OA was diagnosed clinically and radiographically. Bone samples were obtained from the proximal femur during hip replacement surgery (OP and OA groups) or post-mortem (controls). All patients were treatment-naïve, and exclusion criteria were identical across groups, including systemic/metabolic diseases or medications known to affect bone metabolism. OA patients were recruited as a disease-control group to distinguish OP-specific changes from those related to other skeletal pathology. Controls consisted of 10 donors (mean age 68.1 years), in whom skeletal or metabolic disorders were excluded. The demographic and clinical characteristics of all groups, including age, sex, BMI, and BMD parameters, are outlined in Table 1 [33]. Bone and muscle tissue samples were obtained post-mortem from age-matched donors during routine autopsies at the Institute of Forensic Medicine, Faculty of Medicine, University of Ljubljana. Micro-CT scans of bone samples were performed as described previously [32], and the results showed that cadaver bone samples have normal bone structure, confirming that these samples are not osteoporotic and thus suitable as control samples. The study was approved by the Republic of Slovenia National Medical Ethics Committee (Reference Numbers: 0120-523/2016-2, KME 45/10/16). All participants provided written informed consent in accordance with the Declaration of Helsinki.

**Table 1:** The demographic and clinical characteristics of all study groups

	OA (*n*** = **37)	OP (*n*** = **47)	K (*n*** = **10)	*p* (OA–OP)
Age (years)	71.1 (49–87)	76.3 (53–88)	68.1 (56–87)	0.2513
Women	24	38	0	
Men	13	9	10	
BMI (kg/m^2^)	29.3 (22.8–43.7)	25.1 (19.3–33.9)	25.3 (19.0–312)	< 0.001
Hip BMD (g/cm^2^)	0.909 (0.541–1.314)	0.696 (0.402–0.974)	na	< 0.001
Hip *t*-score	− 0.629 (− 3.600 to 1.900)	− 2.261 (− 4.400 to 0.000)	na	< 0.001
Femoral neck BMD (g/cm^2^)	0.791 (0.474–1.179)	0.396 (0.386–0.798)	n**.i**	< 0.001
Femoral neck *t*-score	− 1.192 (− 4.160 to 1.800)	− 2.681 (− 4.200 to − 0.500)	na	< 0.001
Lumbar spine BMD (g/cm^2^)	1.015 (0.583 to 1.401)	0.837 (0.601 to 1.461)	na	< 0.001
Lumbar spine *t*-score	− 0.465 (− 4220 to 2.800)	− 2.006 (− 4.100 to 3.800)	na	< 0.001

### RNA Isolation from Bone and Muscle Tissue

Following sample collection, 200 mg of bone and 100 mg of gluteus medius muscle tissue were immediately snap frozen in liquid nitrogen. Frozen bone and muscle samples were pulverized in liquid nitrogen and homogenized in 1 mL of TRIzol reagent (Invitrogen, Carlsbad, CA, USA). Following the addition of 400 µL chloroform, samples were vigorously mixed and centrifuged at 12,000×*g* for 15 min at 4 °C. The aqueous phase was collected, and total RNA was isolated using the peqGOLD Total RNA Kit (PeqLab, VWR, Germany) as per the manufacturer’s protocol. RNA was resuspended in RNase-free water. RNA concentration and purity were assessed using a NanoDrop spectrophotometer (Thermo Fisher Scientific, USA), and RNA integrity (RIN) was evaluated using the Agilent 2100 Bioanalyzer (Agilent Technologies, Palo Alto, CA, USA).

### Cell Culturing

The human osteosarcoma cell lines HOS (ATCC® CRL-1543™) and MG-63 (ATCC® CRL-1427™) were obtained from the American Type Culture Collection (ATCC, Manassas, VA, USA). Both cell lines were cultured in low-glucose Dulbecco’s modified Eagle medium (DMEM; Gibco, Thermo Fisher Scientific, USA) supplemented with 10% fetal bovine serum (FBS), 2 mM l-glutamine, and 1% antibiotic/antimycotic solution (Gibco). Cells were maintained at 37 °C in a humidified atmosphere containing 5% CO_2_.

### Osteogenic Differentiation of HOS Cells

For osteogenic differentiation, HOS and MG-63 cells were seeded at 5 × 10^4^ cells/cm^2^. After 24 h, differentiation was initiated by replacing the medium with osteogenic induction medium: complete growth medium supplemented with 100 nM dexamethasone, 50 µg/mL 2-phospho-l-ascorbic acid trisodium salt, and 10 mM β-glycerophosphate disodium salt hydrate (all from Sigma-Aldrich, USA). Medium was refreshed every 2–3 days. Non-differentiated control cells were maintained in complete medium for the full duration of the 35-day experiment. Samples were collected at day 0 (prior to induction) and then every 7 days. All experiments were performed in three independent biological replicates.

### Alizarin Red S staining

Mineral deposition was assessed by Alizarin Red S staining as described previously [26]. At each time point, cells were fixed in 4% formalin for 10 min at room temperature, washed with distilled water, and stained with 2% Alizarin Red S solution (pH 4.2; Sigma-Aldrich) for 30 min. After extensive washing, stained cultures were visualized under a light microscope (EVOS XL Core Imaging System, Thermo Fisher Scientific). For quantification, the stain was extracted using 10% (v/v) acetic acid, and absorbance was measured at 405 nm.

### Generation of *MPP7* Knockout HOS Cells

Stable *MPP7* KO HOS and MG-63 cells were generated using CRISPR/Cas9 gene editing. A guide RNA targeting exon 4 of the *MPP7* gene (5′-CCAGCTTTGTCAACGGGATCTGG-3′; Ensembl ENST00000398795, GRCh38.p2) was cloned into the PX459 vector [pSpCas9(BB)-2A-Puro, Addgene #48139; gift from Feng Zhang] [30] using the BbsI restriction site (New England Biolabs, MA, USA). The construct was verified by DNA sequencing (Eurofins Scientific, Luxembourg). Transfection was performed using PolyJet™ In Vitro DNA Transfection Reagent (SignaGen Laboratories, MD, USA), with a PolyJet:DNA ratio of 3:1. After 24 h, cells were selected in 0.5 µg/mL puromycin (Sigma-Aldrich) for 2 weeks. Clonal populations were obtained using cloning rings, and following sufficient expansion, *MPP7* KO was confirmed by Western blotting (Figure S1) as described previously [34]. Briefly, WT and *MPP7*-KO cells were trypsinized, counted and an equal number of cells was lysed and resolved by SDS-PAGE. Primary antibodies against *MPP7* (1:1000 dilution, overnight incubation at 4 °C; Proteintech, Cat# 12983-1-AP, Rosemont, IL, USA) were detected by secondary anti-rabbit antibodies (1:10,000 dilution, 3-h incubation at room temperature; Cell signaling, Cat #7074), glyceraldehyde-3-phosphate dehydrogenase (GAPDH) was used as loading control (1:1000 dilution; rabbit polyclonal, Sigma-Aldrich, #G9545).

### RNA Expression Analysis by RT-qPCR

Total RNA was isolated and cDNA was synthesized with the High-Capacity cDNA Reverse Transcription Kit with RNase Inhibitor (Applied Biosystems, CA, USA) following manufacturers’ instructions. Quantitative PCR (RT-qPCR) was performed using the LightCycler 480 (Roche Diagnostics, Switzerland) with 2.5 ng/µL cDNA and Hot FirePol EvaGreen qPCR Supermix (Solis BioDyne, Estonia) according to manufacturers’ instructions. Cycling conditions were as follows: 95 °C for 15 min (initial denaturation), followed by 40 cycles of 95 °C for 5 s, 60 °C for 20 s, and 72 °C for 20 s. Gene expression was determined for the following markers; *ALPL* (alkaline phosphatase, biomineralization associated), collagen type I alpha 1 chain (*COL1A1*), RUNX family transcription factor 2 (*RUNX2*), osteocalcin (*OC*; bone gamma-carboxyglutamate protein), peroxisome proliferator-activated receptor gamma (*PPARG*), sclerostin (SOST), myogenic differentiation 1 (*MYOD*), and *MPP7*. *EF1A1* and *YWHAZ* were used as reference genes for HOS cells, *TBP* for MG-63 cells [35], and *RPLP0* for patient samples. Primers (Table S1) were designed using NCBI Primer-BLAST and synthesized by Macrogen Europe B.V. (Amsterdam, The Netherlands). Primer efficiency was validated with a standard curve, and specificity was confirmed by melting curve analysis. Gene expression was quantified in duplicate, and relative expression levels were normalized using the ΔΔ*C*_t_ method to the geometric mean of reference genes where applicable.

### Statistical Analyses

Statistical analyses were performed using Prism 7.0 (GraphPad Software, La Jolla, CA, USA). Data are presented as mean ± SEM. Statistical significance was determined using Student’s *t* test, one-way or two-way ANOVA, followed by Bonferroni or Tukey’s post hoc tests. Statistical significance is displayed as follows: ns—not significant (*P* > 0.05); *P ≤ 0.05; **P ≤ 0.01; ***P ≤ 0.001; ****P ≤ 0.0001. *MPP7.*

## Results

### In Silico Prioritization of *MPP7* at the 10p11.23 Locus

**Fig. 1:**
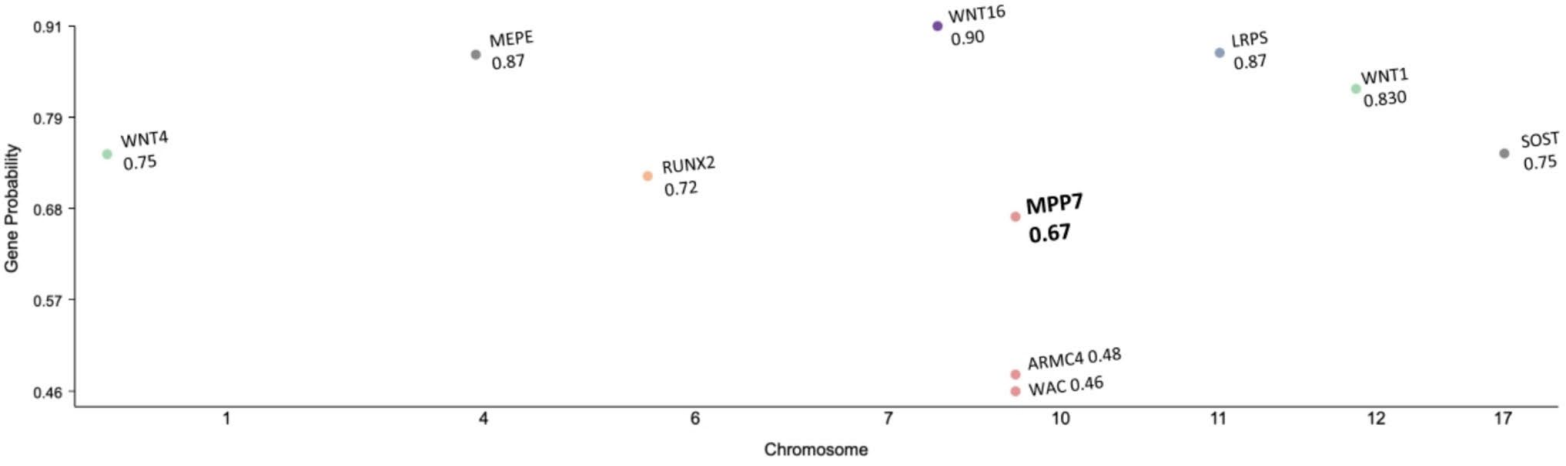
Gene Effector Index prioritization at the 10p11.23 locus. The Gene Effector Index analysis supports *MPP7* as the primary effector gene underlying the GWAS association with lumbar spine BMD, when compared to other screened genes. Expression of *MPP7* is decreased in human osteoporotic bone tissue

To further evaluate the relevance of *MPP7* among candidate genes at the 10p11.23 locus, we performed bioinformatic analyses. Bayesian fine-mapping using the HuGE calculator assigned a Bayes Factor of 45 to *MPP7* for lumbar spine and heel BMD, supporting a high probability of causality at this locus. Consistently, *MPP7* scored significantly for bone tissue expression in Gene Sifter analysis (*P* = 0.0037). Finally, prioritization using the GEI highlighted *MPP7* as the most likely effector gene in this region, with a higher probability of causality than neighboring genes (Fig. 1).

**Fig. 2:**
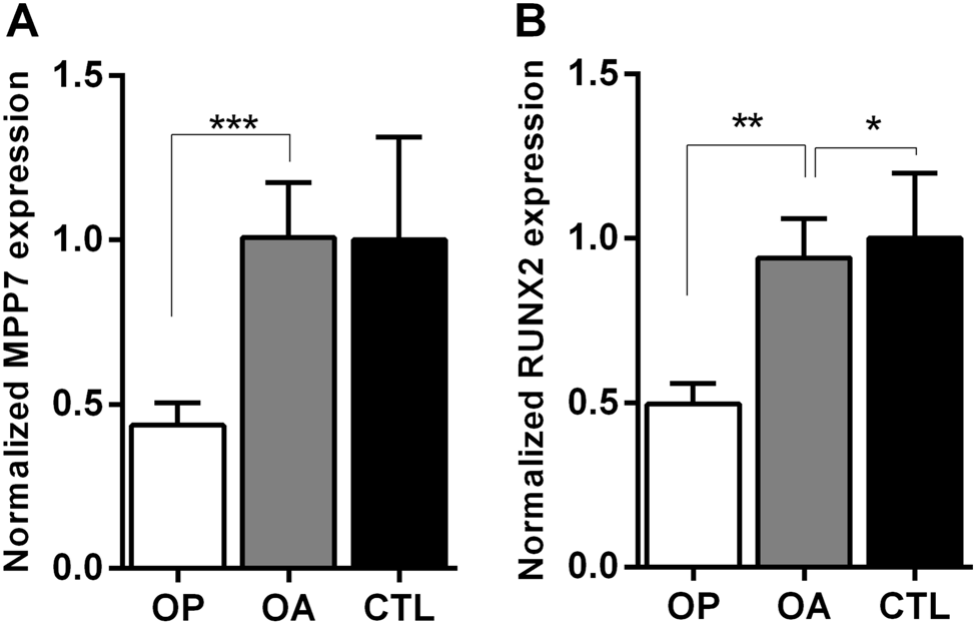
Expression of **A** *MPP7* and **B** *RUNX2* is decreased in osteoporotic (OP) but not osteoarthritic (OA) human bone samples. Gene expression was quantified by RT-qPCR, using *RPLP0* as the internal control. Data are presented as mean fold change ± SEM. Statistical significance was assessed by one-way ANOVA followed by Tukey’s post hoc test, *P < 0.05; **P < 0.01; ***P < 0.001

*MPP7* has been identified as a strong candidate gene in multiple GWAS, examining heel [14, 16–18] and lumbar spine [10, 19] BMD. To assess whether *MPP7* expression is associated with bone pathology, we analyzed its expression in bone tissue samples from individuals with OP, OA, and control donors (healthy cadavers) using RT-qPCR. *MPP7* expression was significantly reduced in OP bone tissue compared to both OA and control (CTL) bone, with a 2.30-fold and 2.28-fold decrease, respectively (*P* = 0.0007; Fig. 2A). Similarly, we observed a significant reduction in the expression of *RUNX2*, a master transcription factor essential for osteoblast differentiation, matrix production, and bone mineralization. *RUNX2* expression was approximately twofold lower in OP samples compared to controls (*P* = 0.002), with a comparable decrease also observed in comparison with OA bone tissue (Fig. 2B), consistent with our previous findings [32]. These results confirm that both *MPP7* and *RUNX2* are reduced in osteoporotic bone and are most probably involved in the pathological processes.

### *MPP7* Expression is Decreased in Osteoporotic Muscle Tissue

**Fig. 3:**
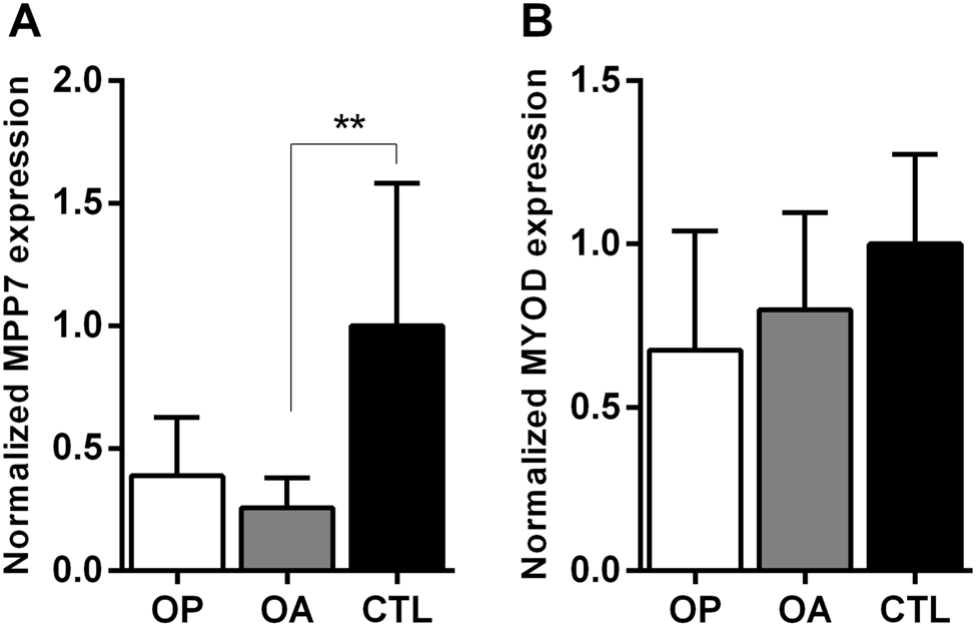
Expression of **A** *MPP7* is significantly decreased in skeletal muscle samples from osteoporotic (OP) and osteoarthritic (OA) patients, whereas **B** *MYOD1* shows only a slight, non-significant reduction compared to controls. Human gluteus medius muscle samples were obtained from patients with OP (*n* = 7), OA (*n* = 11), and healthy autopsy donors (CTL, *n* = 6). Gene expression was measured by RT-qPCR using *RPLP0* as the internal control. Data are presented as mean fold change ± SEM. Statistical significance was assessed by one-way ANOVA with Tukey’s post hoc test,**P < 0.01

Given the close developmental and functional relationship between bone and muscle—mediated through a shared MSC origin and continuous biomechanical and biochemical cross-talk—we next investigated whether *MPP7* gene expression is also altered in skeletal muscle tissue from OP and OA patients. Total RNA was isolated from gluteus medius muscle samples, and the expression of *MPP7* and myogenic differentiation 1 (*MYOD1*) gene was evaluated by RT-qPCR. Interestingly, *MPP7* expression was significantly decreased in both OP and OA muscle tissue compared to controls, with a 2.6-fold and 3.9-fold reduction, respectively (*P* = 0.004; Fig. 3A). In contrast, expression of *MYOD1*, a key transcription factor regulating myogenic differentiation, exhibited only a slight, non-significant decrease. This reduction was more pronounced in OP muscle tissue, but did not reach statistical significance (Fig. 3B). These findings indicate that while *MPP7* expression is reduced in muscle tissue from both OP and OA patients, this downregulation does not appear to significantly affect the transcriptional status of muscle differentiation.

### Deletion of *MPP7* Abolishes Mineralization in Human Osteosarcoma Cells

**Fig. 4:**
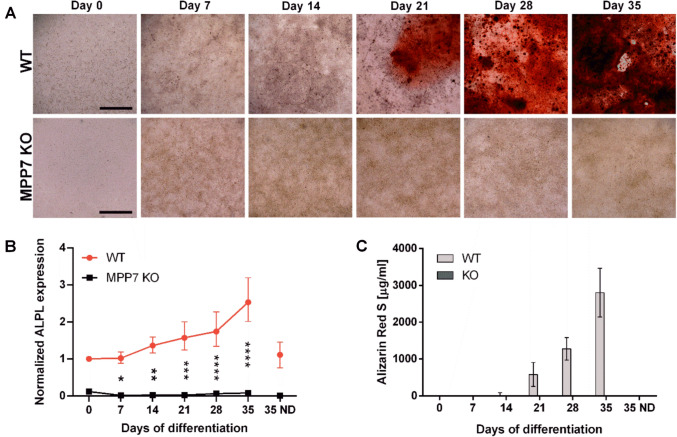
Mineralization and *ALPL* expression are abolished in *MPP7*-KO cells. Wild-type (WT) and *MPP7*-KO HOS cells were cultured in osteogenic differentiation medium and analyzed every 7 days up to day 35. **A** Representative light microscopy images of differentiating WT and *MPP7*-KO cells stained with Alizarin Red S to detect calcium deposits (red). Scale bars represent 1 mm. **B** Relative mRNA expression of alkaline phosphatase (*ALPL*) as determined by RT-qPCR. **C** Quantification of Alizarin Red S staining by spectrophotometry. Data represent mean ± SD of three independent biological replicates (*N* = 3), *P ≤ 0.05; **P ≤ 0.01

Analysis of *MPP7* expression on WT HOS cells showed that *MPP7* mRNA increases during differentiation process up to day 14 and then slowly decreases to day 35, indicating that *MPP7* is involved in osteogenic differentiation (Figure S2). To further confirm the role of *MPP7* in osteogenic differentiation and mineralization, we generated a CRISPR/Cas9-mediated *MPP7* KO in the human osteosarcoma HOS and MG-63 cell lines and assessed their osteogenic potential in vitro. WT and *MPP7*-KO cells were cultured in osteogenic differentiation medium for 35 days. Differentiation was evaluated by measuring the expression of osteogenic markers, while mineralization was assessed both microscopically and spectrophotometrically using Alizarin Red S staining. No mineral deposition was observed in *MPP7*-KO cells throughout the 35-day differentiation period, as demonstrated by the absence of Alizarin Red S staining (Fig. 4A, C for HOS cells and Figure S3, for MG-63 cells). In contrast, WT cells of both cell lines showed detectable mineralization by day 21, with the mineralized matrix progressively expanding to cover the entire cell layer by day 35 (Fig. 4A). These findings indicate that *MPP7* is essential for mineralization in HOS and MG-63 cells. To further characterize the mechanism underlying the mineralization defect, we quantified the expression of *ALPL*, an early osteoblast differentiation marker. In *MPP7*-KO cells, *ALPL* mRNA expression was completely abolished and did not increase at any time point during osteogenic induction (Fig. 4B for HOS and Figure S3B for MG-63). In contrast, HOS WT cells exhibited a gradual increase in *ALPL* expression starting at day 7, reaching a maximum by day 35 (Fig. 4B), while in MG-63, *ALPL* expression peaked at day 7 and gradually decreased till day 35 (Figure S3A). Consistent with its mRNA expression, ALP activity in MG-63 gradually increases till day 21 and then gradually decreases. These results suggest that loss of *MPP7* disrupts the osteogenic mineralization signaling cascade upstream of *ALPL* expression, ultimately leading to a complete block in mineralization.

### Deletion of *MPP7* Affects Expression of Osteogenic Differentiation Markers in Osteosarcoma Cells

**Fig. 5:**
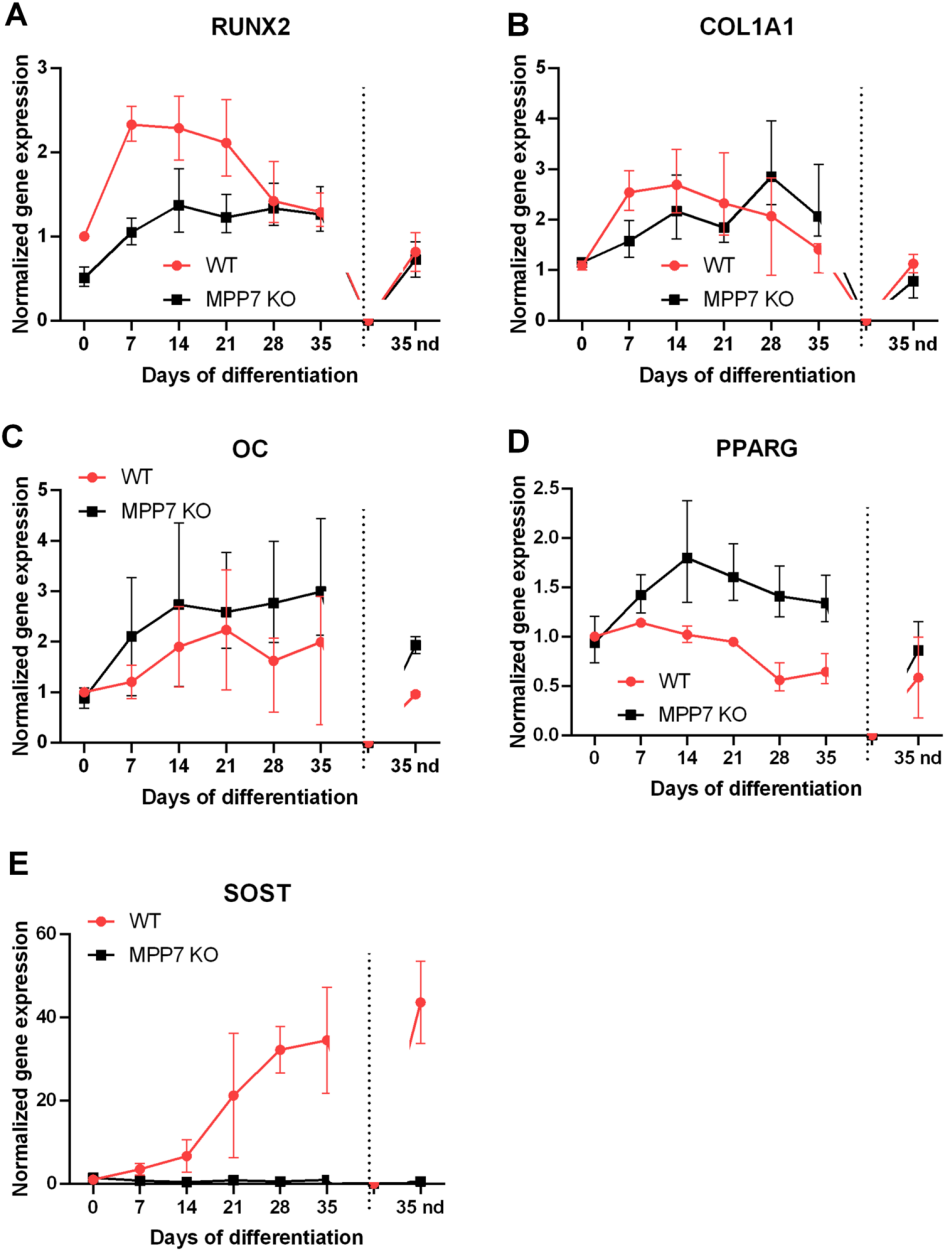
*MPP7* knockout affects the expression of osteogenic and adipogenic differentiation markers. Expression of **A** *RUNX2*, **B** *COL1A1*, **C** *OC* (osteocalcin), **D** *PPARG*, and *SOST* (**E**) was assessed in wild-type (WT) and *MPP7* knockout (*MPP7*-KO) HOS human osteosarcoma cells during 35 days of osteogenic differentiation. Cells were collected weekly (*ND* not determined), and mRNA levels were quantified by RT-qPCR. Expression data were normalized to the geometric mean of *EF1A1* and *YWHAZ* reference genes. Data represent mean ± SEM from three independent biological replicates (*N* = 3), *P ≤ 0.05; **P ≤ 0.01

To identify which osteogenic and functional differentiation markers are affected by *MPP7* deletion, we measured the mRNA expression levels of *RUNX2*, COL1A1, OC, and the adipogenic marker PPARG during the differentiation process. *RUNX2* expression significantly increased in HOS WT cells during the early phase of osteogenic differentiation, peaking at day 7 and gradually returning to baseline by week 3 (Fig. 5A). In contrast, *RUNX2* expression in HOS *MPP7*-KO cells was markedly reduced—2.2-fold lower than in HOS WT cells at day 7—and remained consistently lower throughout the first 21 days of differentiation, increasing only modestly over time. By week 4, *RUNX2* levels were comparable between KO and WT cells. Interestingly, in MG-63 cell line, although *RUNX2* expression increased with time of differentiation in both WT and KO cells, it was significantly higher in MPP-KO cell compared to WT MG-63 cells at all observed time points (Figure S4A). The expression of COL1A1, an early osteogenic marker, followed a similar trend in both cell lines for both WT and KO cells. In HOS cells expression increased until day 14, then gradually declined (Fig. 5B), while in MG-63 cells the highest expression was detected already at day 7, followed by gradual decline, expression in KO cells being slightly higher at all time points (Figure S4B). In *MPP7,* there were no statistically significant differences between KO and WT groups at any time point. Expression of OC, a marker of mature osteoblasts, was also induced during osteogenic differentiation but remained slightly lower in *MPP7*-KO HOS cells compared to WT (Fig. 5C), indicating impaired terminal differentiation, while no differences were detected in MG-63 cells (Figure S4C). In *MPP7*-KO cells, *SOST* mRNA expression, which is an osteocyte marker, was completely abolished and did not increase at any time point during osteogenic differentiation (Fig. 5E). In contrast, HOS WT cells exhibited a gradual increase in *SOST* expression starting at day 14, reaching a maximum by day 35. These results are in line with the previously demonstrated requirement of MPP7 for mineralization as shown by Alizarin Red staining and *ALPL* expression. They suggest that osteocyte formation is impaired, resulting in the absence of SOST expression. Interestingly, PPARG, the master regulator of adipogenic differentiation, showed a significant increase in HOS *MPP7*-KO cells during osteogenic induction (Fig. 5D), while its expression steadily decreased in WT cells, as expected. These findings suggest that loss of *MPP7* alters the expression dynamics of key transcription factors involved in lineage commitment. Reduced *RUNX2* and elevated PPARG expression in *MPP7*-deficient cells may indicate a shift away from the osteogenic toward adipogenic potential.

### Knockout of *MPP7* in HOS Cells Results in Loss of Cell-Layer Morphology

**Fig. 6:**
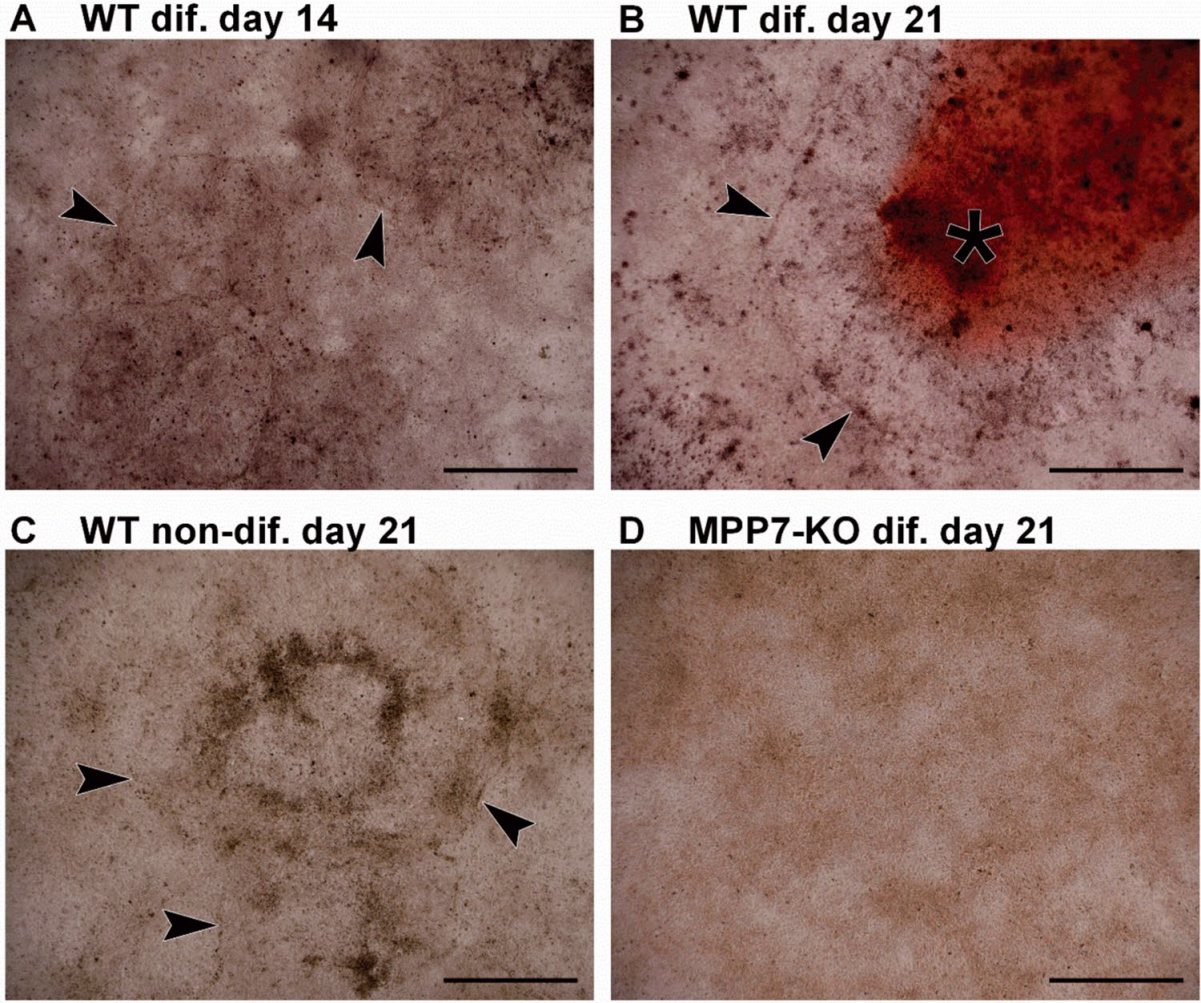
*MPP7* knockout disrupts cell-layer morphology during osteogenic differentiation. Wild-type (WT) and *MPP7*-KO HOS cells were cultured under osteogenic conditions, and morphological changes in the developing cell layer were assessed. Representative images show: **A** WT cells at day 14 of differentiation, **B** WT cells at day 21, **C** non-differentiated WT cells at day 21, and **D** *MPP7*-KO cells at day 21 of differentiation. Arrowheads indicate the margins of circular structures forming within the cell layer, and asterisks denote mineral deposits detected by Alizarin Red S staining. Scale bars represent 1 mm

Microscopic analysis of cultures stained with Alizarin Red S revealed distinct changes in cell-layer morphology during osteogenic differentiation in WT compared to *MPP7*-KO HOS cells, while no changes to cell-layer morphology were observed in MG-63 cells. In HOS WT cells, once confluence was reached, the monolayer began to organize into circular structures composed of margin cells and morphologically distinct central cells. These circular formations progressively increased in number and size over time (Fig. 6A; see also Fig. 4A). By day 21, mineral deposits began to appear in the center of these structures (Fig. 6B), suggesting that they may serve as nucleation sites for mineralization. As mineralization progressed, the deposits expanded to cover the entire cell layer, and the circular structures were no longer discernible. Interestingly, similar circular arrangements were also observed in WT cultures maintained in standard growth medium (i.e., without osteogenic supplements) for the full 35-day period. However, no mineralization occurred in these non-differentiated cultures (Fig. 6C), indicating that the formation of circular structures is a part of normal monolayer development upon confluence but does not lead to mineralization in the absence of osteogenic stimuli. Strikingly, these circular structures were entirely absent in *MPP7*-KO cultures throughout the differentiation period. *MPP7*-deficient cells retained a uniform morphology, and the monolayer remained morphologically homogenous (Fig. 6D; see also Fig. 4A). These observations suggest that *MPP7* contributes to the spatial organization and structural remodeling of the osteoblast monolayer. The inability of *MPP7*-KO cells to form morphologically distinct domains may underlie their failure to initiate mineralization, pointing to a potential role for *MPP7* in orchestrating osteoblast polarity and microenvironmental cues required for matrix deposition.

*MPP7* (MAGUK P55 scaffold protein 7) has emerged as a strong candidate gene for OP in several GWAS [14, 16]. To confirm its direct involvement in bone metabolism, we examined the role of *MPP7* in bone mineralization and OP by analyzing its expression in human bone and muscle tissues and by generating a CRISPR/Cas9-mediated *MPP7* KO in a human osteosarcoma cell model of osteogenesis [36]. Our results demonstrate that *MPP7* expression is significantly reduced in osteoporotic bone tissue (Fig. 2) and that *MPP7* plays a critical role in osteogenic differentiation and mineralization by modulating *ALPL* and *RUNX2* expression (Figs. 4, 5).

**Fig. 7:**
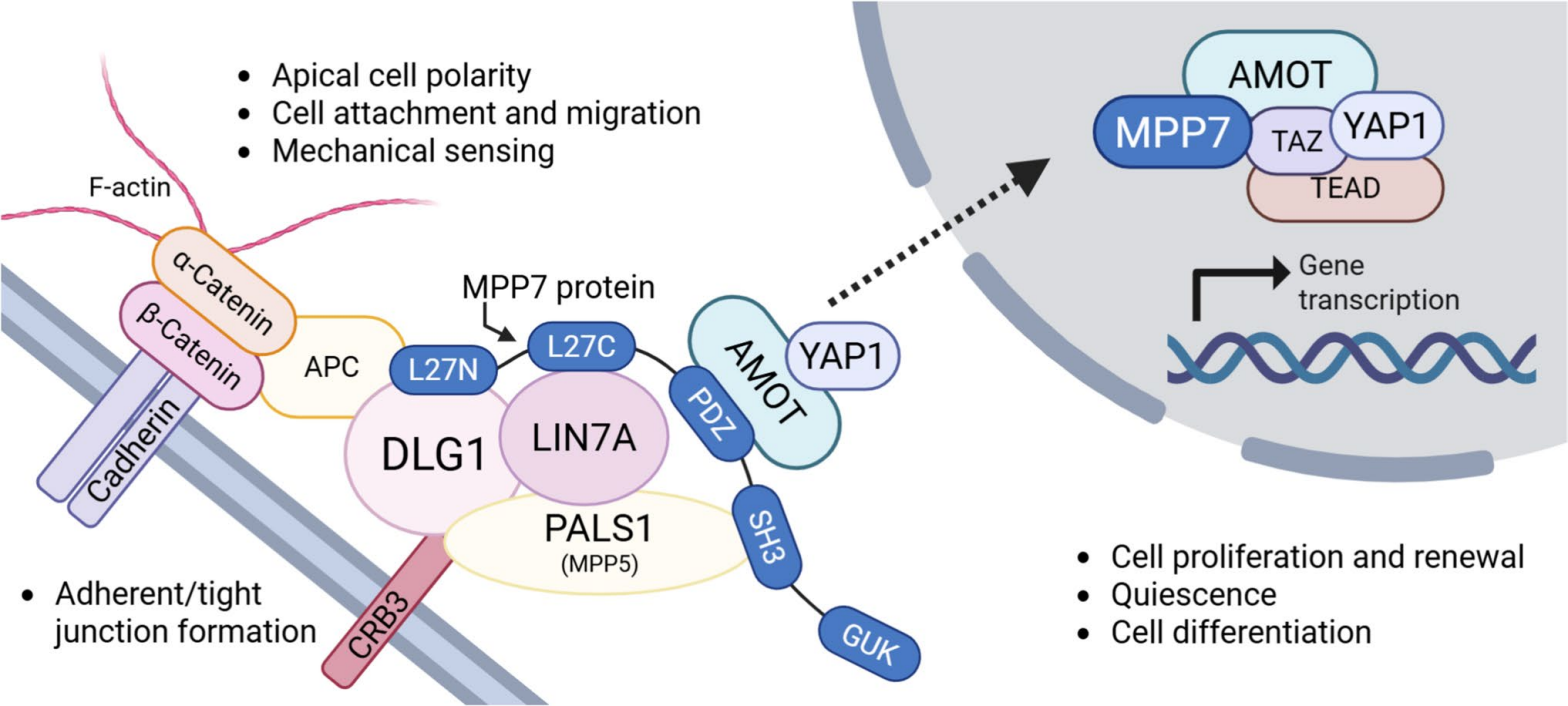
A schematic representation of presumed model of protein interactions of *MPP7*. MPP family proteins are adaptor molecules that assemble protein complexes consisting of protein families such as Crb homologues (e.g., CRB1, CRB3), DLT homologues, and LIN7 homologues (e.g., LIN7A, LIN7B, or LIN7C), together forming a cell adhesion signaling apparatus involved in establishment and maintenance of apical polarity at sites of adherent or tight junctions. *MPP7* protein consists of several domains (i.e., L27N, L27C, PDZ, SH3, and GUK domains) that serve as docking sites for other proteins. While the DLG1–LIN7A–*MPP7* protein complex is mainly involved in stabilization of the protein junctions, it also serves as a docking site for AMOT and YAP1 proteins, forming the *MPP7*–AMOT–YAP1 complex. Based on cell activity and signals form the extracellular environment, this complex can detach and serve as a shuttling complex for YAP1 nuclear entry, where it is also involved in formation of the YAP1/TAZ–TEAD transcriptional regulator complex, which regulates cell growth, proliferation, stem cell renewal, and differentiation. *MPP7* protein is thus involved in establishment and stabilization of cell apical polarity through adherent and tight junctions, as well as in processes of cell proliferation, quiescence, and cell differentiation. *DLG1* disks large homolog 1, *LIN7* Lin-7 homolog, *AMOT* angiomotin, *YAP1* yes-associated protein 1, *TAZ* (also WWTR1)—Tafazzin family protein, *TEAD* TEA domain transcription factor 1, *APC* adenomatous polyposis coli, *CRB3* crumbs cell polarity homolog-3, *PALS1* (also MPP5) protein associated with LIN7 1

Bone remodeling involves a dynamic balance between bone formation and resorption, and *MPP7* may contribute to both processes. Xiao et al. reported increased *MPP7* expression during osteogenesis of human bone-derived cells [21], which was also observed in WT HOS cell line (Figure S2), suggesting a potential role in osteoblast development. To test this directly, we knocked out *MPP7* in HOS and MG-63 cells and found that mineralization was completely abolished in KO cells (Fig. 4), confirming a pivotal role for *MPP7* in the mineralization process (Fig. 4). Mechanistically, *MPP7* may contribute to mineralization by regulating intercellular junctions, Wnt signaling, or both. Previous studies have shown that cell–cell adhesion and junctional integrity are essential for osteoblast differentiation [37, 38]. For instance, inhibition of cadherin-mediated interactions suppresses BMP-2-induced *ALPL* activity and impairs matrix mineralization [39]. *MPP7* has been shown to be essential for tight junction formation [24] and is a component of protein complexes involved in maintaining cell polarity and junctional organization [24, 25, 27] (Fig. 7). Specifically, *MPP7* is part of the Crumbs complex, which plays a crucial role in epithelial polarity [25]. Thus, *MPP7* may influence osteoblast differentiation via structural roles in cell–cell contact formation and polarity establishment [25, 40]. Supporting this hypothesis, *MPP7*-KO HOS cells exhibited altered monolayer morphology and failed to form the circular structures seen in WT HOS cultures—structures that appeared to serve as initiation sites for mineral deposition (Fig. 6). Interestingly, these structures also formed in non-differentiated WT cells cultured in growth medium, suggesting that they arise during confluency and may be required, though not sufficient, for mineralization. No such changes or any cell-layer organization was observed in neither WT nor *MPP7*-KO MG-63 cell line (Figure S3A).

Muscle tissue also plays a crucial role in skeletal health, not only by providing mechanical support but also by sharing the common MSC and cross-communication through both physical and biochemical signals [41]. Our results show that *MPP7* expression is decreased in muscle tissue of OP and OA patients, but it does correlate with *MYOD1*,* the transcription factor* required for muscle cell differentiation. In the study by Andersen et al., polymorphisms in several muscle-related genes were shown to influence bone quality, supporting the concept of genetic interplay between muscle and bone. However, polymorphisms in MYOD1 did not demonstrate any significant association with BMD or fracture risk, suggesting that genetic variation in this myogenic regulator does not directly impact skeletal outcomes [42]. To further investigate the molecular pathways affected by *MPP7* deletion, we analyzed the expression of key osteogenic markers including *COL1A1*, *ALPL*, *OC*, and *RUNX2 and SOST* (Figs. 4, 5). Strikingly, *ALPL* expression was absent in both HOS and MG-63 *MPP7*-KO cells (Fig. 4B), suggesting that *MPP7* acts upstream of *ALPL*. As a critical enzyme for bone mineralization, *ALPL* contributes both to matrix deposition and signaling regulation. Its absence could directly explain the failure of mineralization in *MPP7*-KO cells.

Supporting this, Liu et al. [43] demonstrated that *ALPL* KO in murine osteoblasts downregulates Wnt signaling—a pathway essential for MSC commitment to the osteoblast lineage. Suppressed Wnt activity can lead to a shift from osteogenesis to adipogenesis [44]. In our HOS model, increased expression of *PPARG*, a master regulator of adipogenesis, was observed in *MPP7*-KO cells (Fig. 4D), suggesting a potential lineage shift. However, since Wnt signaling may not be the only affected pathway, additional mechanisms likely contribute. One such pathway may be Hippo signaling, as *MPP7* interacts with AMOT and YAP1 in satellite stem cells to regulate proliferation and regeneration [27, 28] (Fig. 6). A limitation of our study is that HOS and MG-63 cells, although widely used for osteogenesis studies, are already committed osteoblast-like cells of fibroblast/epithelial origin. Thus, this model does not allow direct assessment of lineage commitment. Still, some degree of adipogenic gene expression has been reported in WT HOS cells [45], supporting the relevance of our observations.

Despite the fact that *RUNX2* transcription factor regulates expression of *COL1A1* and *ALPL*, there was no direct correlation between the expressions of these factors. *RUNX2* transcription factor is involved during all stages of osteoblast differentiation, matrix production, and mineralization. Decreased *RUNX2* expression results in abnormal bone development, characterized especially by lower osteoblast differentiation and consequent lower bone ossification [46]. While *RUNX2* was moderately reduced, *COL1A1* expression remained unaffected, and *ALPL* was completely absent (Figs. 3B, 4). This suggests that additional regulatory mechanisms influence the expression of these genes independently of *RUNX2*.

## Conclusion

In conclusion, this study strongly suggests an association between the *MPP7 *gene expression and OP. Importantly, we provide evidence that deletion of *MPP7* hinders mineralization of osteoblasts indicating that *MPP7* has a direct role in bone formation mechanisms. Moreover, we show that *MPP7* is a candidate biomarker for diagnosis of OP. These findings offer new insight into the complex mechanisms of bone pathophysiology and warrant further studies to elucidate the precise molecular pathways by which *MPP7* regulates bone mineralization.

### Potential Limitations of the Study

This study has several limitations. One major limitation is the lack of data on BMD on control (age- and sex-matched healthy cadavers) obtained through DEXA. The quality of the bone was instead confirmed through micro-CT, which showed that control samples have normal bone structure. Where necessary, BMD data from AO patients were used as a phenotypic and mechanistic “control” for OP patients in terms of bone density [47], as studies have shown that OA is often associated with normal or elevated BMD, particularly in subchondral regions [48, 49]. For other analyses, control (cadaver) samples were used.

Another limitation of the study is the low number of collected samples especially in control group, which combined with high heterogeneity of patient samples resulted in low statistical power. The role and effects of *MPP7* should thus be confirmed and further explored on a bigger sample size.

## Supplementary Information

Below is the link to the electronic supplementary material.

Supplementary file1 (DOCX 551 KB)

## Data Availability

The data supporting the findings of this study are available from the first and corresponding author upon reasonable request. No publicly unavailable datasets were generated or analyzed during the current study.

## Declarations

### Permission to Reproduce Material from Other Sources

Not applicable. All figures and content are original.
