# Supplementary material for: Membrane Palmitoylated Protein 7 is Required for Osteogenesis and is Linked with Bone Mineralization and Osteoporosis: The Functional Evaluation of GEFOS GWAS Hit
Source: Calcif Tissue Int. 2025 Oct 17;116(1):128. doi: 10.1007/s00223-025-01439-w (PMC12534256; doi:10.1007/s00223-025-01439-w)
Supplement: Supplementary file 1 — Supplementary file1 (DOCX 551 KB) [file 223_2025_1439_MOESM1_ESM.docx]

**Membrane palmitoylated protein 7 is required for osteogenesis and is linked with bone mineralization and osteoporosis - The Functional Evaluation of GEFOS GWAS hit**

Petra Malavašič^1,2^, Jasna Lojk^3^, Nika Lovšin^2^, Radko Komadina^4^, Gregor Haring^5^, Rihard Trebše^6,7^, Fernando Rivadeneira^8^, David Karasik^9^, Barbara Ostanek^2^, Janja Marc^2,3^*

^1^ Novo mesto General Hospital, Dept. of Laboratory Diagnostics, Novo mesto, Slovenia

^2^ University of Ljubljana, Faculty of Pharmacy, Ljubljana, Slovenia

^3^University Medical Centre Ljubljana, Clinical Institute of Clinical Chemistry and Biochemistry, Ljubljana, Slovenia

^4^ General Hospital Celje, Department for Research and Education, Celje, Slovenia

^5^ University of Ljubljana, Institute of Forensic Medicine, Ljubljana, Slovenia

^6^ Orthopaedic Hospital Valdoltra, Ankaran, Slovenia

^7^ University of Ljubljana, Faculty of Medicine, Ljubljana, Slovenia

^8^ Erasmus University Medical Center, Rotterdam, Netherlands

^9^ Bar-Ilan University, The Azrieli Faculty of Medicine, Safed, Israel

**Supplementary information**


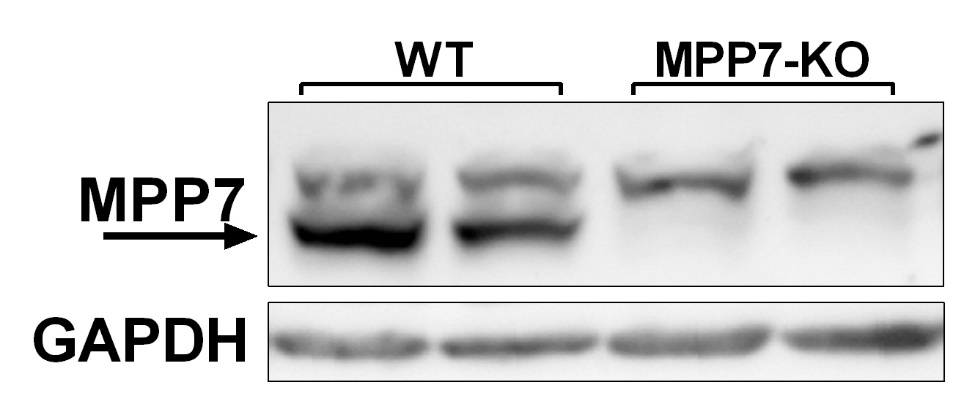


**Figure S1:** **CRIPSR-Cas9 knock-out of MPP7 gene in HOS osteosarcoma cell line results in total absence of MPP7 protein** as demonstrated with Western Blot. GAPDH was used as loading control. Representative blots are shown.

**Table 1:** Primer sequences used in this study.

| **Symbol** | **Gene name** | **Primer sequence** |
| --- | --- | --- |
| ALPL | Alkaline phosphatase, biomineralization associated | F: 5’- CCAAGTACTGGCGAGACCAA-3’  R: 5’- GTGGAGACACCCATCCCATC-3’ |
| COL1A1 | Collagen type I alpha 1 chain | F: 5’- GCCAAGACGAAGACATCCCA - 3’  R: 5’- GTTTCCACACGTCTCGGTCA - 3’ |
| RUNX2 | RUNX family transcription factor 2 | F: 5’- AGCAAGGTTCAACGATCTGAGAT - 3’  R: 5’- TTTGTGAAGACGGTTATGGTCAA - 3’ |
| PPARG | Peroxisome Proliferator Activated Receptor Gamma | F: 5’- CACAAGAACAGATCCAGTGGTTGCAG- 3’  R: 5’- AATAATAAGGTGGAGATGCAGGCTCC- 3’ |
| OC | Osteocalcin (Bone Gamma-Carboxyglutamate Protein) | F: 5’- AAGAGACCCAGGCGCTACCT- 3’  R: 5’- AACTCGTCACAGTCCGGATTG- 3’ |
| MPP7 | MAGUK P55 Scaffold Protein 7 | F: 5’- TTATACCCGGCAGCAAAGAG- 3’  R: 5’- TGAGGCTGAACATCCAACAA- 3’ |
| MYOD | Myogenic Differentiation 1 | F: 5’- TGCCACAACGGACGACTTC- 3’  R: 5’- CGGGTCCAGGTCTTCGAA- 3’ |
| RPLP0 | Ribosomal Protein Lateral Stalk Subunit P0 | F: 5’- TCTACAACCCTGAAGTGCTTGAT - 3’  R: 5’- CAATCTGCAGACAGACACTGG - 3’ |
| EF1A1 | Eukaryotic Translation Elongation Factor 1 Alpha 1 | F: 5’- CTGGACTGCATCCTACCACC - 3’  R: 5’- CTCGGCCAACAGGAACAGTA - 3’ |
| YWHAZ | Tyrosine 3-monooxygenase/ tryptophan 5-monooxygenase activation protein zeta | F: 5’- TGCTTGCATCCCACAGACTA - 3’  R: 5’- AGGCAGACAATGACAGACCA - 3’ |
| SOST | Sclerostin | F: 5’- TGGCAGGCGTTCAAGAATGA  R: 5’- TGTACTCGGACACGTCTTTGG |





**Figure S2: Expression of MPP7 changes during HOS osteosarcoma cell line differentiation**. WT HOS human osteosarcoma cells were exposed to osteogenic medium and expression of MPP7 mRNA was determined every 7 days with RT-qPCR.


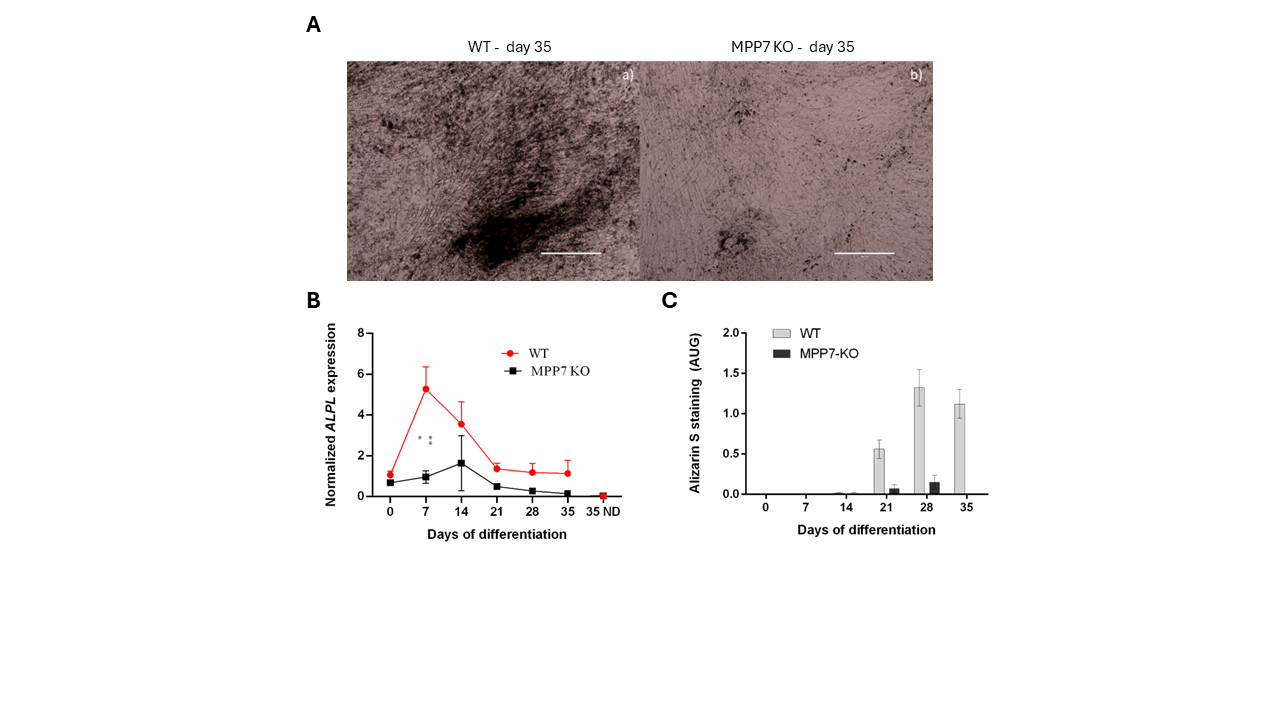


**Figure S3:** **Mineralization and *ALPL* expression are abolished in** *MPP7***-KO MG63 cells.** Wild-type (WT) and *MPP7*-KO MG-63 cells were cultured in osteogenic differentiation medium and analyzed every 7 days up to day 35. (A) Representative light microscopy images of day 35 of differentiating WT and *MPP7*-KO cells. Scale bars represent 1 mm. (B) Relative mRNA expression of alkaline phosphatase (*ALPL*) as determined by RT-qPCR. (C) Quantification of Alizarin Red S staining by spectrophotometry. Data represent mean ± SD of three independent biological replicates (*N* = 3), *P ≤ 0.05; **P ≤ 0.01


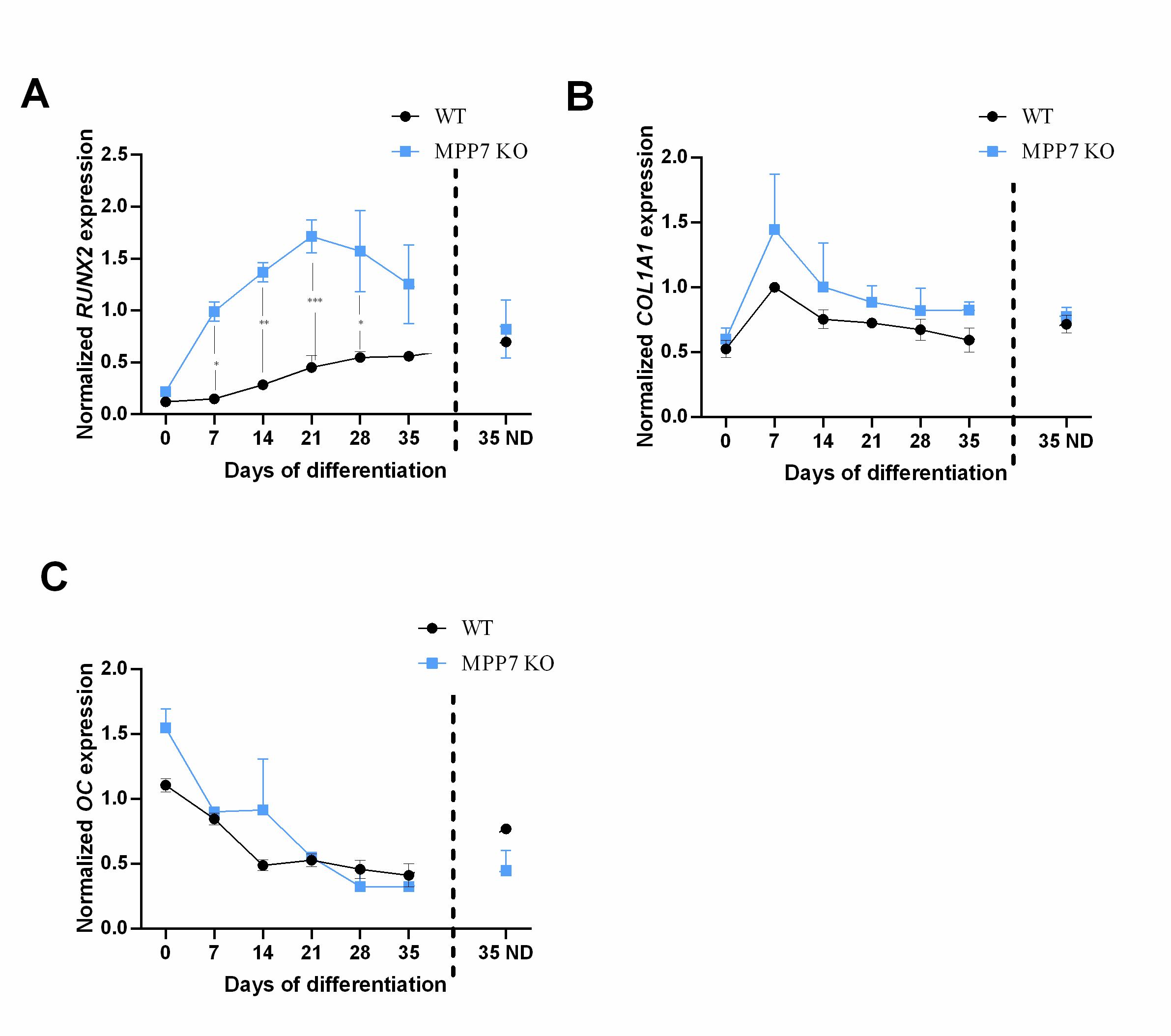


**Figure S4. *MPP7* knockout in MG63 affects the expression of osteogenic differentiation markers** . Expression of (A) *RUNX2*, (B) *COL1A1*, (C) *OC* (osteocalcin) was assessed in wild-type (WT) and *MPP7*-knockout (MPP7-KO) MG-63 human osteosarcoma cells during 35 days of osteogenic differentiation. Cells were collected weekly (ND = not determined) and mRNA levels were quantified by RT-qPCR. Expression data were normalized to the geometric mean of *EF1A1* and *YWHAZ* reference genes. Data represent mean ± SEM from three independent biological replicates (*N* = 3), *P ≤ 0.05; **P ≤ 0.01
